# Role of Glycogenolysis in Memory and Learning: Regulation by Noradrenaline, Serotonin and ATP

**DOI:** 10.3389/fnint.2015.00070

**Published:** 2016-01-19

**Authors:** Marie E. Gibbs

**Affiliations:** Drug Discovery Biology, Monash Institute of Pharmaceutical Sciences, Monash University, ParkvilleVIC, Australia

**Keywords:** astrocytes, glycogen re-synthesis, noradrenaline, ATP, serotonin, memory processing, consolidation, day-old chickens

## Abstract

This paper reviews the role played by glycogen breakdown (glycogenolysis) and glycogen re-synthesis in memory processing in two different chick brain regions, (1) the hippocampus and (2) the avian equivalent of the mammalian cortex, the intermediate medial mesopallium (IMM). Memory processing is regulated by the neuromodulators noradrenaline and serotonin soon after training glycogen breakdown and re-synthesis. In day-old domestic chicks, memory formation is dependent on the breakdown of glycogen (glycogenolysis) at three specific times during the first 60 min after learning (around 2.5, 30, and 55 min). The chicks learn to discriminate in a single trial between beads of two colors and tastes. Inhibition of glycogen breakdown by the inhibitor of glycogen phosphorylase 1,4-dideoxy-1,4-imino-D-arabinitol (DAB) given at specific times prior to the formation of long-term memory prevents memory forming. Noradrenergic stimulation of cultured chicken astrocytes by a selective β_2_-adrenergic (AR) agonist reduces glycogen levels and we believe that *in vivo* this triggers memory consolidation at the second stage of glycogenolysis. Serotonin acting at 5-HT_2B_ receptors acts on the first stage, but not on the second. We have shown that noradrenaline, acting via post-synaptic α_2_-ARs, is also responsible for the synthesis of glycogen and our experiments suggest that there is a readily accessible labile pool of glycogen in astrocytes which is depleted within 10 min if glycogen synthesis is inhibited. Endogenous ATP promotion of memory consolidation at 2.5 and 30 min is also dependent on glycogen breakdown. ATP acts at P_2_Y_1_ receptors and the action of thrombin suggests that it causes the release of internal calcium ([Ca^2+^]_i_) in astrocytes. Glutamate and GABA, the primary neurotransmitters in the brain, cannot be synthesized in neurons *de novo* and neurons rely on astrocytic glutamate synthesis, requiring glycogenolysis.

## Introduction

The major fuel for all cells in the brain is glucose and both astrocytes and neurons metabolize glucose via glycolysis and oxidative phosphorylation. Neuronal transmission of information is undoubtedly important for memory but astrocytes are also critically involved in memory processing. Memory is dependent on metabolic functions including Na/K-ATPase activity and glucose uptake, which occur in both neurons and astrocytes. It is also reliant on glycogen breakdown and re-synthesis. It is recognized that glycogen has an important role in the brain (e.g., Brown, 2004; Brown and Ransom, 2007; Hertz et al., 2007; Oz et al., 2007; Morgenthaler et al., 2009). Normally only astrocytes can store glucose as glycogen. Glycogen in the brain is stable and has slow turnover under resting conditions (Watanabe and Passonneau, 1973; Choi et al., 2003; Oz et al., 2007). Glycogen is unlikely to be acting solely as a slowly available energy source. The breakdown of glycogen increases substantially during sensory activation in the brain (Swanson, 1992; Swanson et al., 1992; Cruz and Dienel, 2002) and it appears to have an active role in astrocytic function. It is necessary for memory formation in both chickens and rodents (O’Dowd et al., 1994; Gibbs et al., 2006; Suzuki et al., 2011; Newman et al., 2011; Duran et al., 2013). Both Suzuki et al. and Newman et al. assumed that glycogen-derived lactate is used as a metabolic fuel by neurons after release from astrocytes via the astrocytic monocarboxylate transporters MCT 1 and 4 and uptake in neurons via the neuronal MCT 2 (i.e., operation of the astrocyte-to-neuron lactate shuttle (ANLS)) suggested by Pellerin et al. (1998) and Pellerin and Magistretti (2012). However, a recent study by Tadi et al. (2015) showed that learning in mice increased expression of MCT 1 and 4 without affecting MCT 2. Thus astrocytic lactate release is important for learning but it is doubtful whether any neuronal uptake occurs. Moreover convincing evidence has recently been obtained for activity-dependent glucose phosphorylation in neurons (Patel et al., 2014), which focuses attention on recently discovered signaling functions of extracellular lactate (Tang et al., 2014; Bergersen, 2015). In this context it is interesting that the lactate signaling demonstrated by Tang et al. (2014) like memory in the chicken is abolished not only by the glycogenolysis inhibitor DAB (see below) but also by D-lactate (Gibbs and Hertz, 2008). The action of these and other drugs used in the studies to be reviewed are summarized in **Table 1**.

**Table 1:** Individual effects of drugs on glycogenolysis and memory in imm and hippocampus (Hp).

Receptor agonists		
ARC239	α_2C_-AR agonist Activates glycogen synthesis	IMM and HP
RO363	β_2_-AR agonist consolidates ITMA	HP not IMM
Zinterol	β_2_-AR agonist consolidates ITMB Activates glycogenolysis	IMM and HP
CL316243	β_3_-AR agonist consolidates ITMA	IMM and HP
5-HT	Serotonin, an agonist on many subtypes, activates glycogenolysis, but probably not glycogen synthesis	IMM, HP unknown
Fluoxetine	5-HT_2B_ agonist	IMM, HP unknown
Paroxetine	5-HT_2B_ agonist	IMM, HP unknown
ATP	ATP receptor agonist, promotes consolidation 2.5 and 30 and 35 min after training	IMM and HP
ADP| 3S	P2Y1 receptor agonist, promotes consolidation 2.5 and 35 min after training	HP
ATPγS	P2Y2 receptor agonist, promotes consolidation 2.5 and 30 min after training	HP
Thrombin	Stimulates release of internal calcium ([Ca^2+^]_i_) in astrocytes, promotes consolidation 2.5 and 35 min after training	IMM and HP
Phaclofen	GABA_B_ receptor antagonist inhibits memory during STM and ITMA injected up to 25 min after training	IMM and HP

**Receptor antagonists**		

CGP20712A	β_1_-ARs, antagonist inhibits STM	HP not IMM
ICI118551	β_2_-AR antagonist inhibits ITMB	IMM and HP
SR59230A	β_3_-AR antagonist inhibits ITMA	IMM and HP
SB221284	5-HT_2B/C_ R antagonist inhibits STM and ITMB	IMM HP unknown
MRS2179	P2Y1 receptor antagonist, inhibits consolidation 2.5 and 35 min after training	STM and HP
D-AVP	NMDA receptor antagonist, inhibits 2.5 and 30 min after training	IMM and HP

**Metabolic inhibitors**		

DAB	Inhibits glycogenolysis	IMM and HP
Fluoroacetate	Inhibits astrocytic oxidative metabolism and memory during STM and ITMA injected up to 20 min after training	IMM and HP

## Memory Formation in the Chicken Following One-Trial Discriminated Avoidance Learning

**FIGURE 1:**
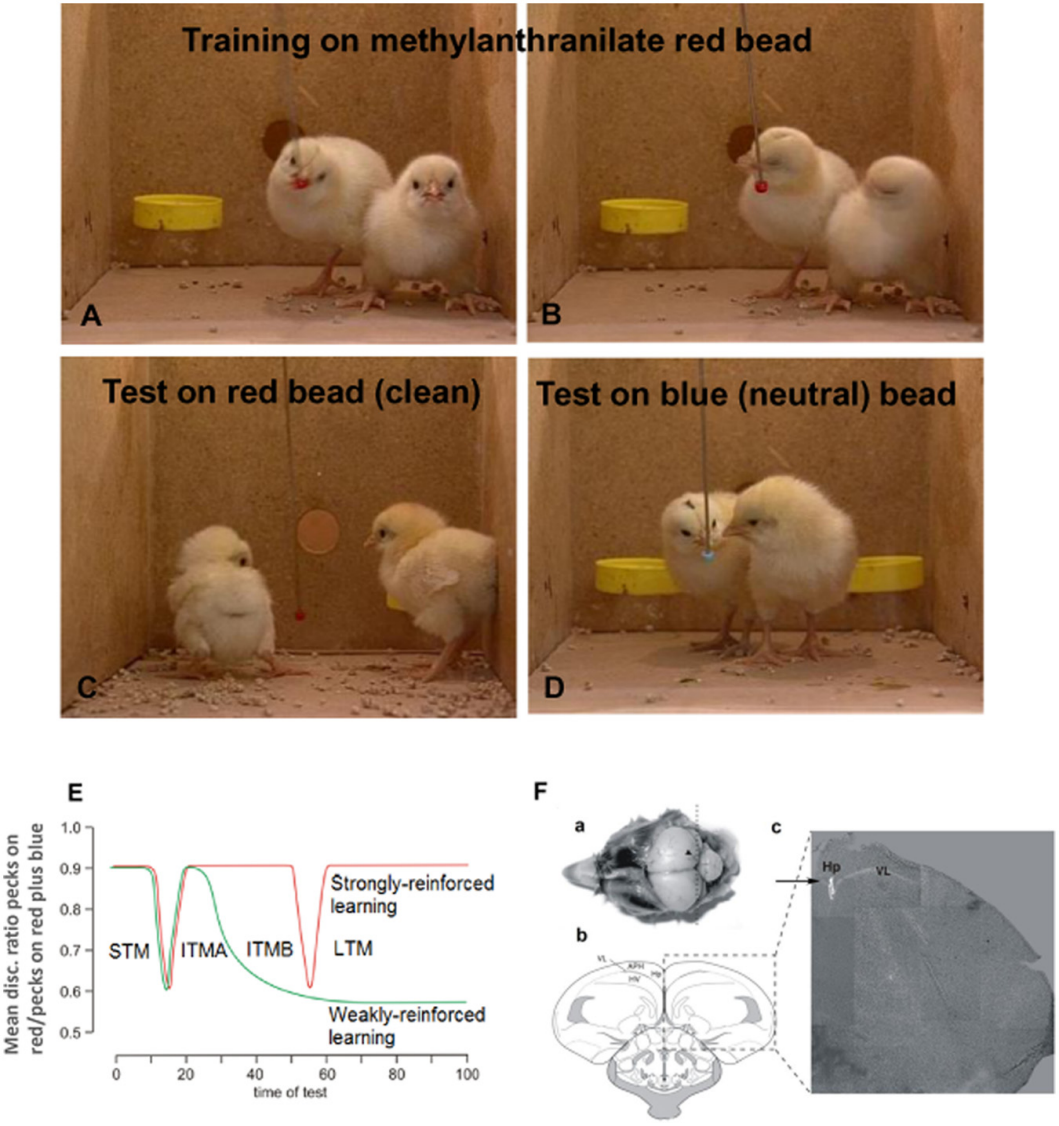
**Memory model established from single trial learning in day-old chicks.** Prior to training chicks are presented once with clean red and blue beads to ensure they will peck at beads of both colors. For training they are presented for 10 sec with a red bead lightly coated in methyl anthranilate **(A)**. They peck at this once or twice before registering the bitter taste and turning away in disgust **(B)**. On test they are then presented with *clean* red **(C)** and blue **(D)** beads, each for 10 s and the number of pecks on each counted using an electronic counter and converted by computer to discrimination ratios (DRs). Perfect learning equals a DR of 1, and complete forgetting or inhibition of learning a DR of 0.5. Normally the DR after unimpaired learning is ∼0.9. The chicks are kept in pairs and 8–10 pairs are included in the group used in each experiment, which allows reliable determination of significance. Each data point on subsequent graphs represents one group. **(E)** Memory stages following strongly reinforced (red line) after exposure to undiluted aversant and weakly reinforced training (green line) after exposure to diluted aversant. The loss of labile, weakly reinforced memory coincides with the transition between two phases of intermediate memory (ITM A and B) 30 min post-training. Drugs are used to inhibit strongly reinforced learning or rescue weakly reinforced learning, as indicated by memory retained 120 min after training. **(F)** Illustration of injection for hippocampal administration of drugs. (a) Image of head with scull removed and injection site indicated by arrowhead. Dotted line indicates coronal section presented in panels (b) and (c). see Gibbs et al., 2008a for details. (1F from Gibbs et al., 2008a).

The experiments described in this review use avoidance training in the day-old chick. Chicks are precocious and can discriminate between colors soon after hatch, and they do so after a single 10 s learning experience. At training a red bead is tainted with a compound of bitter taste (methyl anthranilate) and presented to the chick for 10 s. They peck at this bead once or twice (**Figure 1A**) before registering the bitter taste and turning away in disgust (**Figure 1B**). On test after a predetermined interval they are then presented with *clean* red (**Figure 1C**) and blue (**Figure 1D**) beads, each for 10 s. The chickens will avoid the second (untainted) red bead when presented, but they will continue to peck when presented with a neutral blue bead. Memory is measured as the ratio of pecks at red and blue beads on test. A high discrimination ratio reflects a good memory and avoidance of pecking at the red bead, whereas a discrimination ratio close to 0.5 reflects an increased rate of pecking at the red bead- such that red and blue beads are pecked at equally on the 10 s test (for details see Gibbs and Summers, 2002a; Gibbs et al., 2008d). It is not uncommon for chicks to give up to 10 pecks at the blue bead, and when they have forgotten they can give up to 10 pecks on the clean red bead.

The chicks are kept in pairs and used in groups of 20, or more recently, in groups of 16 chicks. The data from chicks not pecking the blue bead on test or not pecking the red bead on training are excluded from the data analysis at the completion of the experiment. Each data point on the behavioral graphs represents a single group of chicks with generally no more than 2–3 chicks excluded on the basis on not training or avoiding the blue bead, i.e., *N* ≥ 13–14 when the original number in the group was 16. The very short training period enables exact timing of biochemical events correlated with learning.

The bead is either strongly aversive, i.e., the bitter taste is caused by application of 100% anthranilate, or weakly aversive when anthranilate is diluted to 20% with alcohol. When separate groups of chicks are tested at defined periods post-training after strongly reinforced training three stages of memory are revealed, and the memory remains for days, indicating that this corresponds to normal learning. However, even in normal learning there are brief periods of low discrimination ratios measured on testing at 15 or at 55 min. With weak reinforcement, with the exception of the test at 15 min after training, the memory is good on tests up to 30 min after which memory disappears (**Figure 1E**).

Based on the demonstration of different biochemical events (Gibbs and Ng, 1977) we have defined three stages of memory consolidation during normal learning : short-term (STM)-lasting for 10 min intermediate term memory (ITM), which has two different stages, ITMA between 20 and 30 min and ITMB between 30 and 50 min. The transition is normally triggered by neuromodulatory transmitters, including noradrenaline and serotonin, as will be reviewed here. Weakly reinforced learning (**Figure 1E**) includes ITMA but not ITMB, another indication that these two stages depend upon different metabolic events. The advantage of studying weakly reinforced learning is that it can be converted to normal learning by different procedures, providing information on possible underlying mechanisms. Normal learning can on the other hand be disrupted by other interventions, again providing clues about the underlying events. Accordingly, both types of learning will be discussed in this review.

Drugs are injected into the hippocampus in 1 μl volumes (Gibbs et al., 2008a; **Figure 1F**) or in 5 or 10 μl volumes into the intermediate medial mesopallium (IMM), a cortical integration area in the avian brain serving the same functions as the cortex in mammals (Puelles et al., 2000; Reiner et al., 2004; Jarvis et al., 2005). This area of the brain has been traditionally used for studies on memory (see Gibbs, 2008; Gibbs et al., 2008d). By injecting different groups of chicks at different times, we have been able to determine when memory is vulnerable to either inhibition in the case of strongly reinforced training or consolidation in the case of weakly reinforced training. When injections of inhibitory agents are made after strongly reinforced training, memory is poor on test either at 120 min or at specified times after training; consolidation of weakly reinforced training maintains memory after ITMA. **Figure 1F** shows details of the injection procedure for hippocampal injections and it can be seen that tissue damage due to the injection is minimal (Gibbs et al., 2008a). All procedures reviewed here have been approved by the Monash University Animal Ethics Committee and comply with the 1997 Australian Code of Practice for the care and use of animals for scientific purposes. All efforts were made to minimize both the suffering and the number of animals used. Chicks were killed at the completion of each experiment by CO_2_ inhalation.

**FIGURE 2:**
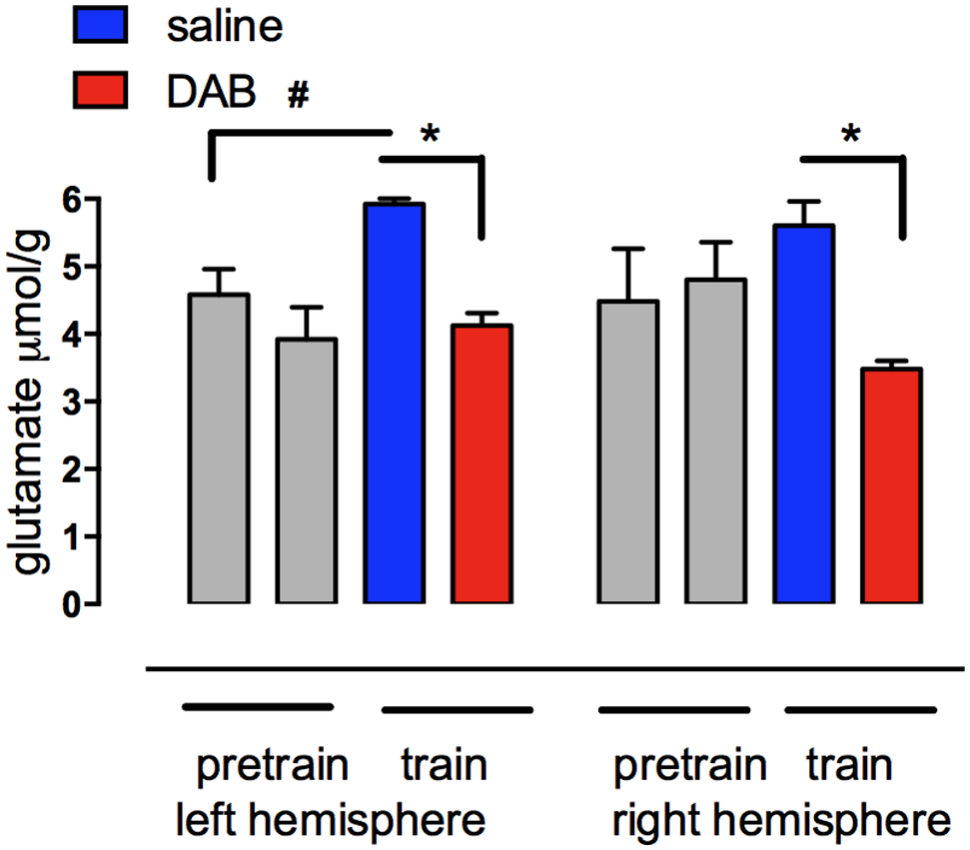
**Glutamate content measured with HPLC in left and right forebrain hemispheres of chicks before and after training in the presence of DAB or saline.** Chicks were injected with either saline or DAB 5 min before strongly reinforced training and brains rapidly removed 5 min after training. The increase in glutamate in the left hemisphere is prevented by DAB. No concomitant decrease occurred in aspartate or glutamine. ^#,^*P < 0.05. (From Gibbs et al., 2007).

Key points of our findings are (1) the involvement of glycogen in memory processing and in synthesis of glutamate, a neurotransmitter essential for learning (Riedel et al., 2003) and long-term potentiation, LTP (Bashir et al., 1993), (2) the role of neuromodulators in glycogenolysis, glycogen synthesis and learning, and (3) mechanisms by which the neuromodulators influence glycogen storage and breakdown and thus the processes during learning which are glycogenolysis-dependent. A crucial role of glycogenolysis in glutamate synthesis (**Figure 2**) was first shown in chick brain during learning (Gibbs et al., 2007) and its role in support of glutamatergic transmission was confirmed in co-cultures of neurons and astrocytes (Sickmann et al., 2009) and in the mouse brain (Sickmann et al., 2012). That synthesis of transmitter glutamate as well as return of previously released transmitter glutamate depends on astrocytic metabolism has been known for a long time (reviewed by Gibbs et al., 2008b; Hertz, 2013; Hertz and Rothman, 2015), however that the astrocytic-neuronal flux carrying glutamate from astrocytes to neurons equals the rate of total neuronal glucose consumption (or 75% of total gray matter oxygen uptake) has only been established more recently (Sibson et al., 1998; Rothman et al., 2011). This shows the intensity of the astrocytic-neuronal interactions required during learning.

## Memory Formation Requires the Breakdown of Glycogen

**FIGURE 3:**
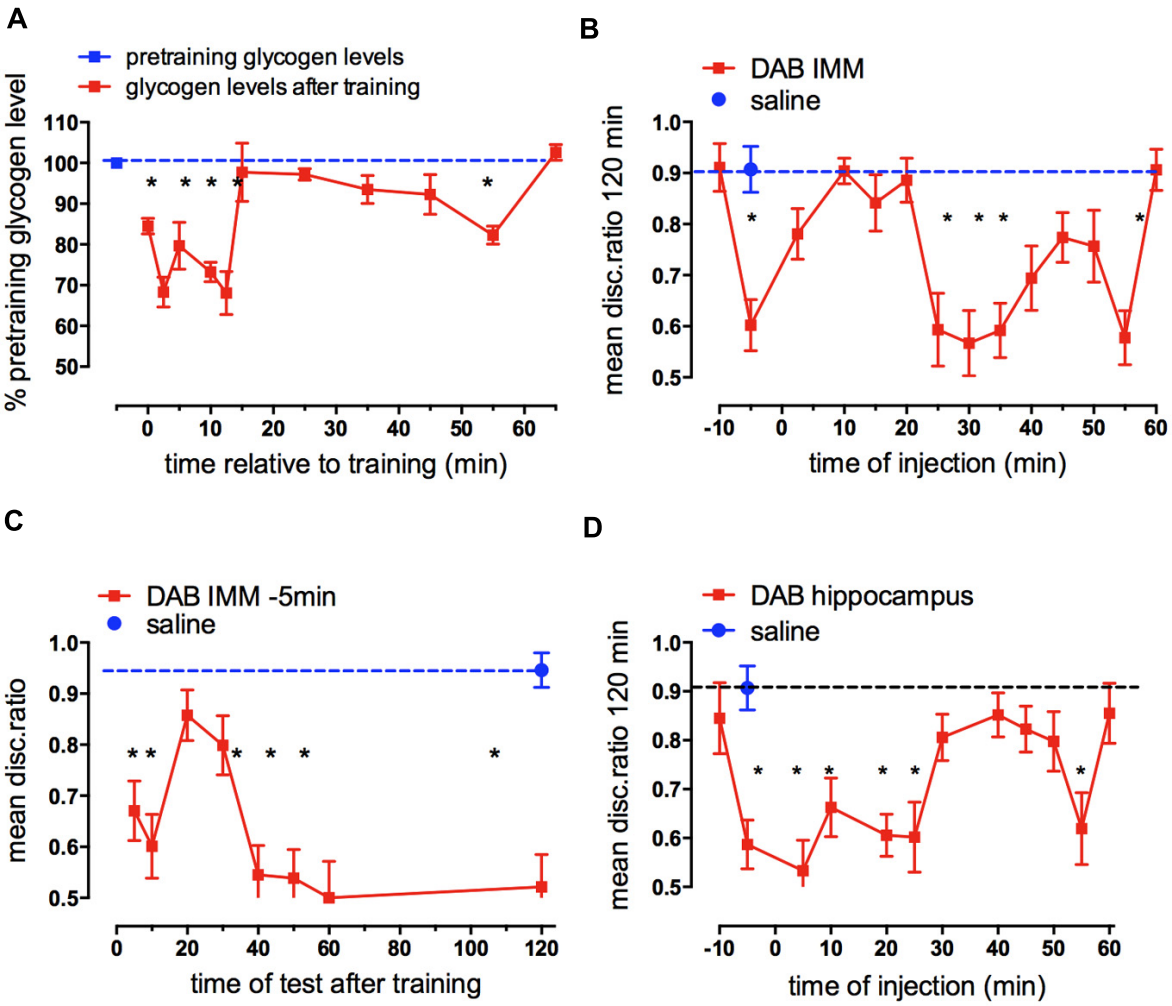
**Glycogen levels after training in chick forebrain and effect on memory consolidation of inhibition of glycogen breakdown. (A)** Glycogen levels in combined left and right hemispheres after training compared with the glycogen content at pre-training. Glycogen was measured as described by Lo et al. (1970) with minor modifications. Brains were excised and transferred to pre-weighted tubes and their weight determined by the increase in weight. After digestion of the tissue, glycogen was precipitated by the addition of 95% ethanol, hydrolyzed to glucose in a phenol-sulphuric acid solution and the absorbance measured at 490 nm and calibrated by aid of a standard glucose curve (From O’Dowd et al., 1994; Hertz et al., 2003). **(B)** Times of injection of DAB into IMM after strongly reinforced training showing those times at which memory processing requires glycogen breakdown; **(C)** Times of test after strongly reinforced training following DAB injection 5 min before training revealing when memory is affected by DAB; **(D)** Times of injection of DAB into the hippocampus following strongly reinforced training. (From Gibbs et al., 2007, 2008b). In this figure and all subsequent graphs the dashed line represents saline control value for strongly reinforced training (high DR) or weakly reinforced training (DR approaching 0.5) ^*^P < 0.05.

Measurement of the change in glycogen levels, expressed as % of pretraining levels revealed two periods where total forebrain levels decreased and later rose to former pretraining levels (O’Dowd, et al., 1994; Hertz, et al., 2003). There is a large, rapid decrease 2.5 min after training where glycogen levels remain low for 10 min and then return to previous levels within 5 min (**Figure 3A**). There is a second transient decrease at 55 min. This fits wth the turnover of brain glycogen increasing during activation of brain tissue (Swanson et al., 1992; Dienel et al., 2003, 2007). A third tendency toward a decrease at 30 min is not significant.

The importance of the breakdown of glycogen for learning is seen when an inhibitor of glycogenolysis or glycogen breakdown is injected into the forebrain at different times after training. The glycogen phosphorylase inhibitor DAB (Andersen et al., 1999; Fosgerau et al., 2000) blocks glycogenolysis but is known not to affect glycolysis.

In chicks where DAB was injected into IMM 5 min before training or 55 min after training, there was very poor memory 2 h later, but injection between 25 and 45 min after training also decreased memory (**Figure 3B**). When does this memory deficit occur? The early blockade of glycogenolysis 5 min prior to training leads to an inability to retrieve memory from short-term storage until after 10 min after training, with no amnestic effect being seen between 20 and 30 min (**Figure 3C**); we concluded therefore that memory retrieval throughout the first part of intermediate memory (ITMA) does not depends on glycogenolysis, but does so on tests from 40 min on. Effects occur early and some of the consequences appear during STM, not ITMA but later during ITMB. However, DAB has effects on memory beyond this time. When memory is monitored after injections made 25 min after training, memory loss is seen at the earliest test made 35 min after training, i.e., 10 min after injection and with injections 55 min after training, memory is lost 15 min later – the earliest time tested (Gibbs et al., 2006).

DAB injected into the hippocampus also leads to memory loss (Gibbs et al., 2008a). However, the pattern of susceptibility in the hippocampus is not not the same as in IMM (see **Figure 3D**). There are three important times where there is coincidence – 5, 25, and 55 min post- training, but there are differences between the two brain areas. DAB inhibits in the hippocampus when injected during ITMA in contrast to IMM, whereas during ITMB (30–50 min after training) DAB is inhibitory in the cortex but not in the hippocampus. When DAB is injected into the hippocampus 5 min before training, memory is present tested at 20 min but as with IMM injection memory is absent when chicks are tested 5 and 120 min after training (Gibbs et al., 2008a). DAB does not appear to produce a long acting inhibition of glycogen phosphorylase, since when it is injected 10 min before training it has no effect on memory. These data and results mentioned below suggest that there is a time lag of 5–10 min before glycogenolysis is significantly inhibited.

The breakdown of glycogen is clearly important for learning. Two of the susceptible periods when long-term memory is prevented occur when DAB is administered during STM (0–10 min post-training) or at the end of intermediate memory (ITMB) 55 min after training, and both periods correlate with the changes in glycogen levels in the forebrain. However, there is a second period when memory is sensitive to DAB (around 20–25 min post-training) but is not associated with a significant decrease in glycogen content, suggesting that glycogen synthesis may be going on at this time, offsetting the inhibitory effect on glycogenolysis. This is supported by the observation that an inhibitor of glycogen synthesis inhibits memory 10–20 and 40–60 min after training (see below).

What is the trigger for glycogenolysis? Evidence is presented here for the involvement of noradrenaline, serotonin and ATP in the breakdown of glycogen for memory processing.

## Noradrenergic Trigger of Glycogenolysis

There is evidence that glycogenolysis is stimulated by noradrenaline (Magistretti, 1988; Quach et al., 1988; Subbarao and Hertz, 1990), and in the chicken, via the β_2_ -adrenergic receptor (Gibbs et al., 2006) at the time of transition between the first and second phases of intermediate memory, i.e., around 25–30 min after training. Noradrenaline acts on two α and three β receptor subtypes, and in extensive studies we have shown that it can do so via all five subtypes (Gibbs and Summers, 2002a, 2005) with effects differentiated by time and brain location.

**FIGURE 4:**
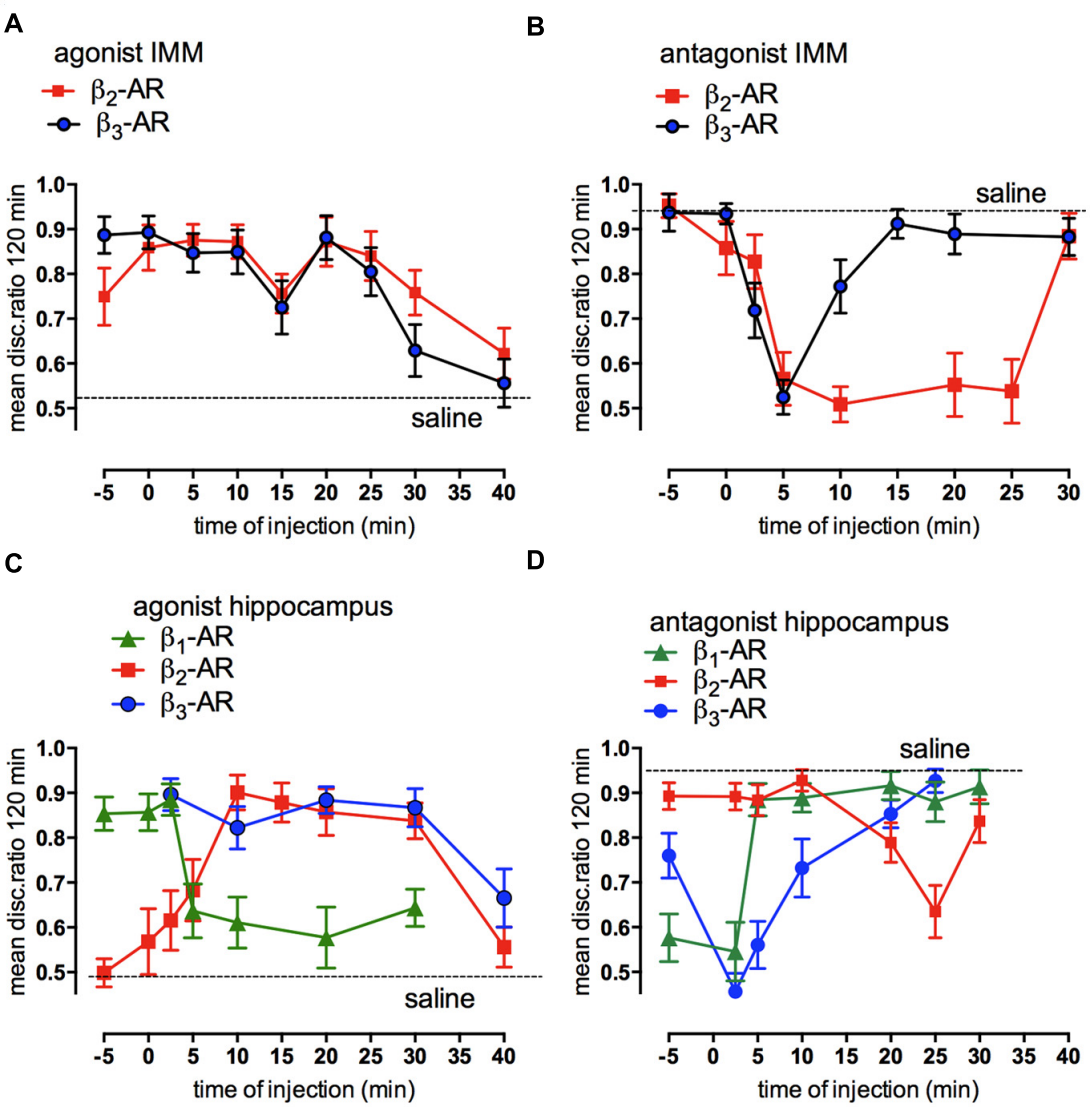
**Involvement of β-adrenoceptors in weakly- and strongly reinforced memory in both avian cortex (IMM) and hippocampus.** Injections of selective β-AR agonists at different times after weakly reinforced training in IMM **(A)** or hippocampus **(C)** and of selective antagonists for β-ARs in IMM **(B)** or hippocampus **(D)** after strongly reinforced learning. Memory tested 120 min after training. (From Gibbs, 2008).

Injected into the cortex, noradrenaline promotes formation of memory after weakly- reinforced training by activation of β_2_- and β_3_-adrenergic receptors as well as by α_1_-ARs. Consolidation of weakly reinforced training is achieved with injection of either β_2_- (zinterol) or β_3_-AR (CL316243) receptor agonists (**Figure 4A**). Both agonists consolidate when injected at any time up to 30 min after training with the exception of a lesser effect with injection given at 15 min post-training. In the avian brain there is a predominance of β_2_-ARs (see Gibbs et al., 2008a), whereas in the mammalian brain mainly the β_1_- subtype of the receptor is expressed (Quach et al., 1978). However, the β_1_- AR agonist RO363 was unable to promote consolidation at any of the times injected into IMM (Gibbs and Summers, 2002a) but there are β_1_- AR effects on memory in other brain areas such as the Medial Striatum and the hippocampus (see below). The specific antagonist for the β_2_-AR (ICI 118551) and for the β_3_-AR (SR59230) both inhibited strongly reinforced learning when injected 5 min after training, with the β_2_-AR antagonist inhibiting up to 25 min after training, i.e., over the duration of ITMA, but memory was spared when the antagonists were given 5 min before training (**Figure 4B**).

In the hippocampus injection of the β-AR agonists promoted consolidation of weakly reinforced training; zinterol and CL316243 both promoted consolidation although with slightly different timing compared to that seen in the IMM. In addition, the β_1_-AR agonist RO363 also promoted consolidation during STM and the antagonist CGP20712A inhibited strongly reinforced training over the same time course (**Figures 4C,D**). Varying the time of injection clearly shows that both the agonists and antagonists had different memory response patterns in the hippocampus and the IMM.

Although the β_2_-AR agonist in IMM can consolidate memory injected up to 25 min post-training (**Figure 4A**), the time at which the antagonist is effective has a close correlation with the time when DAB is ineffective (**Figure 3B**). In the hippocampus glycogenolysis is critical for underwriting the STM and ITMA memory periods as well as at 55 min post-training (**Figure 3D**). The β_2_-AR agonist was only effective for a short time when injected during ITMA, whereas the β_3_-ARs were active during STM and ITMA. The β_1_-AR agonists promoted consolidation with injection during STM only (**Figure 4C**).

**FIGURE 5:**
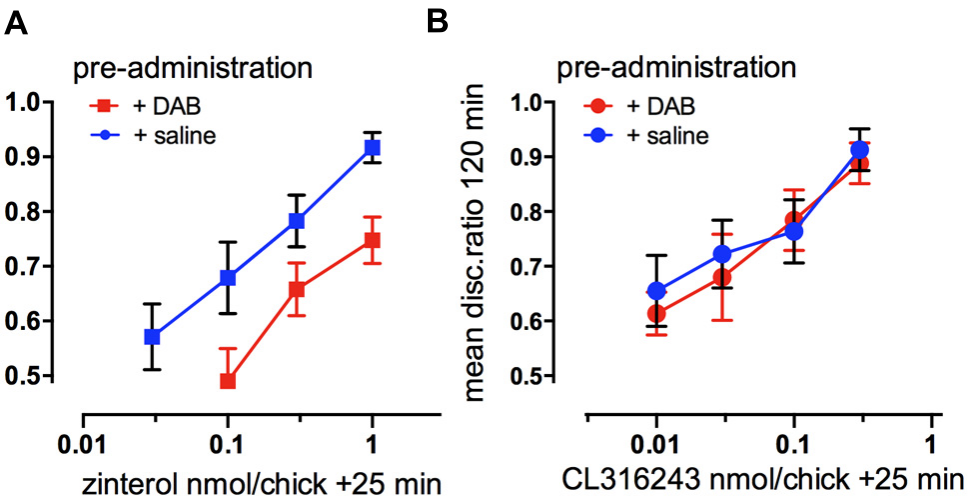
**Functional selectivity of drug interaction. (A)** Consolidation of memory for weakly reinforced training with the β_2_-AR agonist zinterol or **(B)** the β_3_-AR agonist CL316243, challenged by preadministration at 20 min of a suboptimal dose of DAB. Zinterol in the presence of DAB, inhibiting the breakdown of glycogen, was less effective in promoting memory consolidation. (From Gibbs et al., 2008c).

Behavioral experiments to confirm that glycogenolysis is associated with β_2_-adrenoceptors involved challenging the selective agonists with a weak dose of DAB or saline. A dose level of DAB that was not sufficient to block memory itself was injected at a time when it has been shown not to affect memory. In the cortical area (IMM) β_2_- and β_3_-AR agonists were injected 25 min after weakly reinforced training when they normally promote consolidation. DAB reduced the ability of zinterol to consolidate memory (**Figure 5A**), but had no effect on the ability of the CL316243 to consolidate memory (**Figure 5B**). In the hippocampus, a low dose of DAB did not affect the ability of CL316243 or the β_1_-AR agonist RO363 to consolidate memory, but as in IMM, DAB reduced the ability of zinterol to promote consolidation (Gibbs et al., 2008a). Since DAB prevents activation-induced glycogen breakdown and decreases memory, we conclude that glycogen breakdown achieved by stimulation of β_2_-ARs is necessary for memory.

## Glycogen Content and Synthesis in Cultured Chicken Astrocytes

**FIGURE 6:**
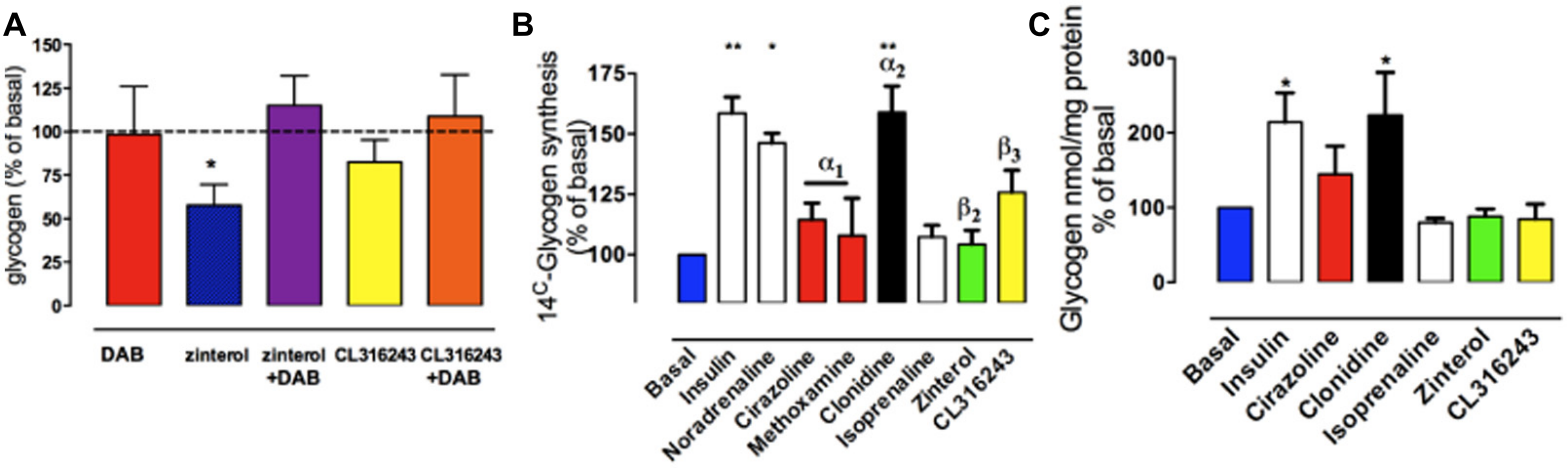
**The effect of adrenoceptor agonists on glycogen levels turnover and new synthesis in cultured astrocytes from chick forebrain. (A)** Astrocytes were incubated for 2 h under basal conditions with no drug added, or for 2 h in the presence of zinterol or CL316243. DAB was added 20 min prior to the 2 h incubation (adapted from from Gibbs et al., 2006). **(B)** Glycogen synthesis assessed by [^14^C]-glucose incorporation into glycogen in response to 3 hr stimulation with AR agonists, noradrenaline or insulin. **(C)** Glycogen levels expressed as % of basal after incubation with AR agonists or insulin. ^*^P < 0.05; ^**^P < 0.001. (From Hutchinson et al., 2011).

The effects on glycogen levels measured in chick astrocyte primary cultures, in the presence of DAB or in the presence of zinterol (with DAB or saline added 20 min prior to zinterol exposure) are shown in **Figure 6A**. As can be seen DAB did not alter the basal glycogen levels but it did prevent the zinterol-induced decrease in glycogen level (Gibbs et al., 2006) but had no significant effect in the presence of the β_3_-adrenergic antagonist CL316243.

If stimulation of noradrenergic β_2_-ARs stimulates memory consolidation via the breakdown of glycogen with no dramatic decrease in glycogen levels (at 30 min post-training), it is likely that there is some cellular mechanism to stimulate the synthesis of glycogen. This increased synthesis is achieved by noradrenaline released from neurons stimulating astrocytic α_2_-ARs (post-junctional receptors; Hertz et al., 2007; Hutchinson et al., 2011; Gibbs and Hutchinson, 2012). At the same time β_3_-adrenergic stimulation increased uptake of glucose, securing the glucose presence needed for the synthesis of glycogen (Gibbs and Summers, 2002a,b; Gibbs et al., 2008c).

**FIGURE 7:**
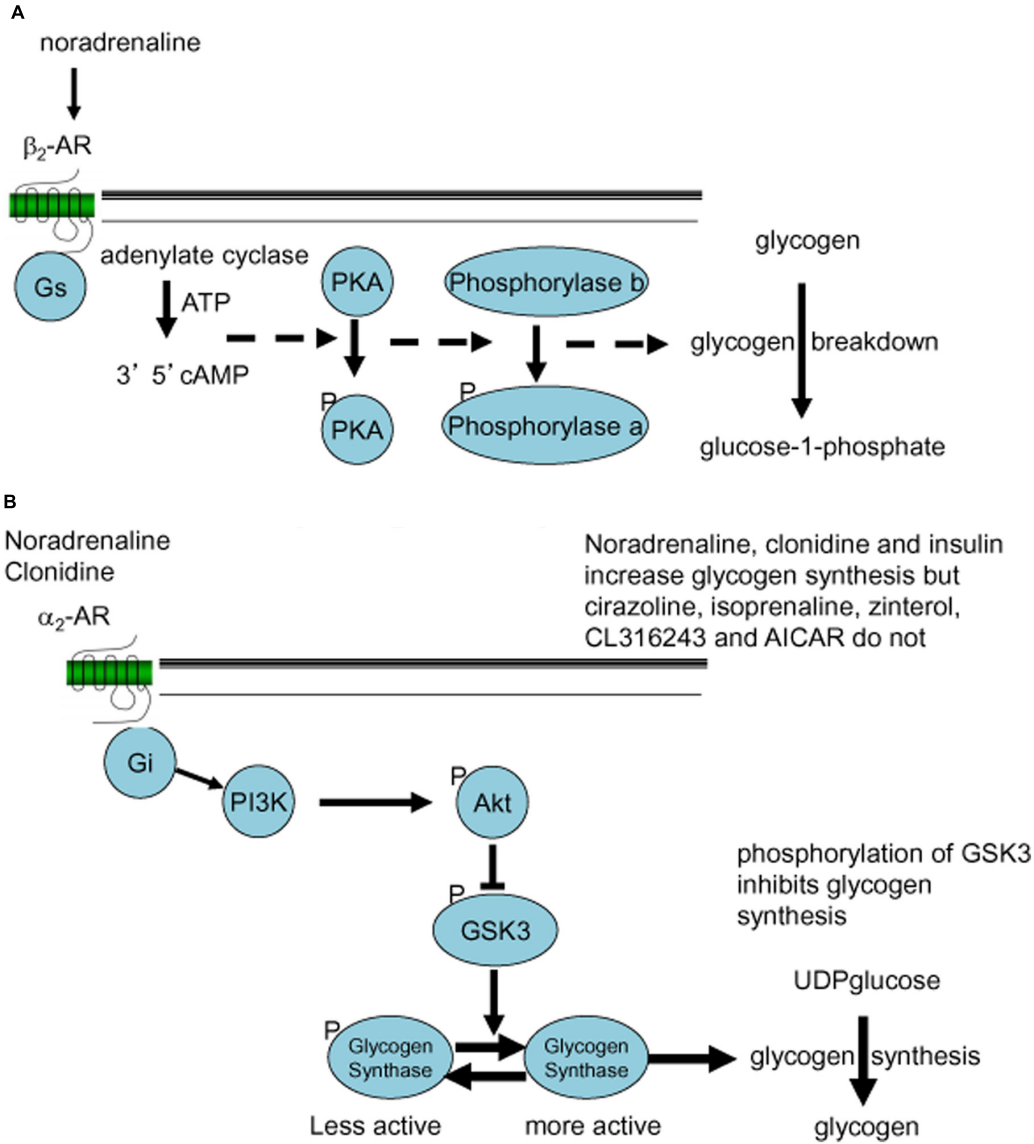
**Noradrenergic signaling stimulating glycogenolysis **(A)** or glycogen synthesis **(B)**. (A)** β_2_-Adrenergic G_s_-mediated formation of cAMP and phosphorylation of proteinkinase A (PKA) leads to conversion (phosphorylation) of phosphorylase b to the active phosphorylase a, which stimulates glycogen breakdown (conversion of glycogen to glucose-1-phosphate). **(B)** α_2_-Adrenergic G_i_-mediated stimulation of the PI3K-AKT pathway leads to phosphorylation of glycogen synthase kinase (GSK) and dephosphorylation of glycogen synthase, which stimulates glycogen synthesis (incorporation of UDPglucose into glycogen).

Noradrenaline increased glycogen formation (total ^14^C incorporation) in astrocytic culture (Hutchinson et al., 2008, 2011). This was not inhibited by the β_1_-/β_2_-AR antagonist propranolol nor by the β_3_-AR antagonist SR59230A, suggesting that the effect of noradrenaline to increase glycogen turnover is not mediated by β-ARs (Hutchinson et al., 2011). Insulin was used as the positive control (**Figure 6B**). When the effects on glycogen levels were compared, insulin and clonidine significantly increased glycogen levels but the other agonists had no effect (**Figure 6C**). A timecourse experiment with noradrenaline, isoprenaline and zinterol demonstrated significantly decreased glycogen levels following 5 and 10 min of stimulation (Hutchinson et al., 2008). In experiments where noradrenaline and subtype selective agonists were incubated with astrocytes, only noradrenaline and the α_2_-AR agonist clonidine increased glycogen synthesis measured as total ^14^C incorporation (% of basal) into glycogen, whereas the β-AR agonists had no significant effect. Noradrenaline stimulates glycogenolysis and formation of cAMP in similar cultures of astrocytes (O’Dowd et al., 1995), consistent with the glycogenolytic mechanism shown in **Figure 7A**.

The α_2_-AR agonist clonidine activates both α_2A_- and α_2C_-ARs and both are present in areas of the brain important for memory processing (Scheinin et al., 1994; Hutchinson et al., 2011). AR α_2A_-and α_2C_-AR subtypes are found both pre and post-synaptically on neurons as well as post-junctionally on astrocytes (Enkvist et al., 1989; Aoki, 1992; Lee et al., 1998a,b; Hertz et al., 2010). In chick astrocytes clonidine stimulation of glycogen synthesis was blocked by the α_2C_-AR subtype selective agonist ARC239 (Gibbs and Hutchinson, 2012). α_2_-Adrenergic stimulation activates glycogen synthesis by stimulation of the AKT pathway and subsequent down-regulation of GSK (**Figure 7B**). This is consistent with the observations that the potent α_2_-AR agonist, dexmedetomidine induces ERK phosphorylation both in cultured astrocytes and in brain slices, but not in cultured neurons (Li et al., 2008; Du et al., 2009), and that the ERK and AKT pathways interact in astrocytes (Dai et al., 2013).

## Effect Of ARC239 Inhibition of α_2C_ Ars on Memory Processing and Comparison with Dab Timecourse

**FIGURE 8:**
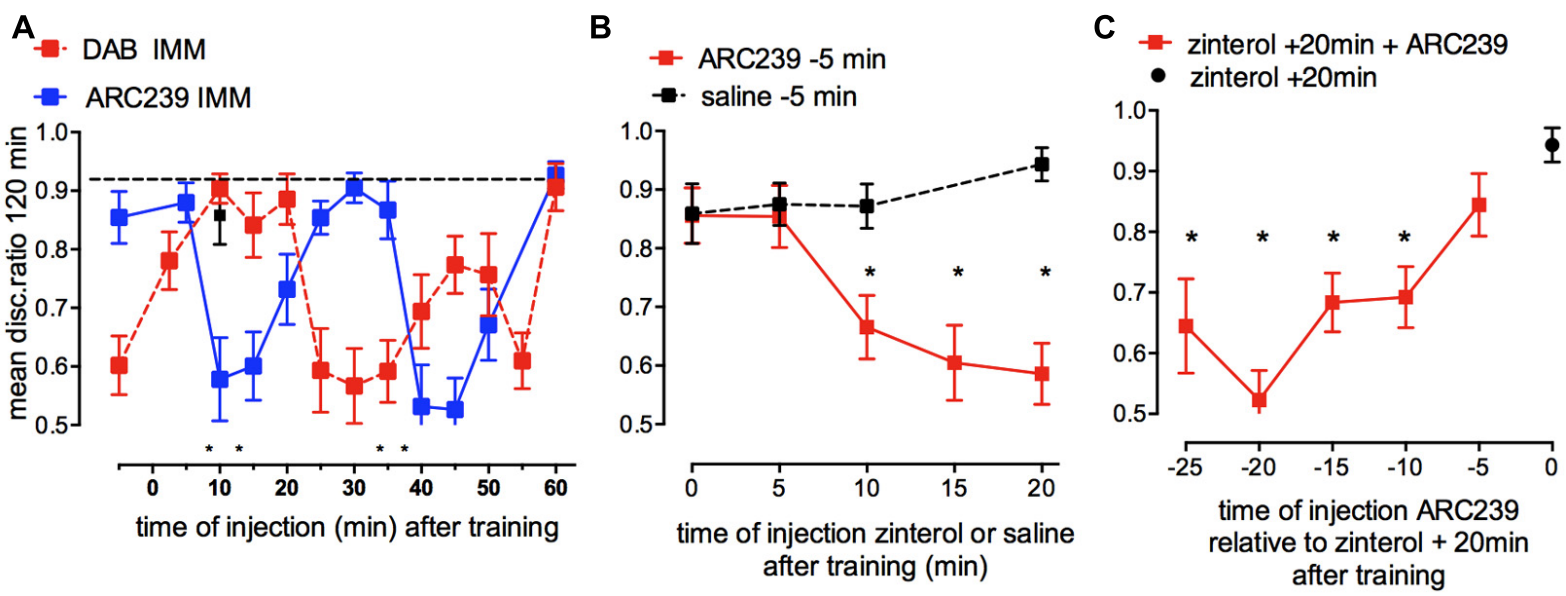
**Effect of inhibition of glycogen re-synthesis by the α_2B/C_-AR antagonist ARC239 on strongly reinforced memory and challenges to inhibition of re-synthesis by β_2_-AR stimulation (glycogen breakdown) and vice versa. (A)** ARC239 was injected into IMM at different times after training. The timing of the effect of ARC239 is compared with that of DAB (From Gibbs et al., 2008b; Hertz and Gibbs, 2009). **(B)** ARC239 or saline were injected 5 min before weakly reinforced training and zinterol injected into different groups at various times after training. Inhibiting re-synthesis of glycogen prevented β_2_-AR stimulation from promoting memory consolidation up to 10 min prior to zinterol injection. Normal learning is indicated by black squares. **(C)** Zinterol was injected into all groups 20 min after weakly reinforced training and ARC239 had been injected at times from 25 to 5 min before zinterol (i.e., from 5 min before to 15 min after training). ^*^P < 0.05. (From Gibbs and Hutchinson, 2012).

Inhibition of the synthesis of new glycogen, caused by injection of the selective α_2C_-AR antagonist ARC239 into IMM revealed a clear time-dependence. Memory at 120 min was inhibited by ARC239 injected at two time periods after training, (i) when injected 10–20 and (ii) when injected 40–50 min after strongly reinforced training (**Figure 8A**). On the other hand, preventing glycogen breakdown with DAB, inhibited memory when injected at three different times: 5 min before, 25–35 min after and at 55 min after training in IMM. The effects of ARC239 and DAB mirror each other *but* with a lag time of about 10 min (Hertz and Gibbs, 2009). ARC239 prevented memory at quite discrete time points, which suggests that its inhibitory action is not sustained for long periods in the brain. In the presence of ARC239 there is insufficient accessible glycogen, so β_2_-ARs although activated cannot induce glycogenolysis and promote consolidation. Since noradrenaline will activate α_2_-AR as well as β_2_-ARs at the same time, any measurement of glycogen levels will reflect the net balance between synthesis and degradation.

There is a slow turnover of glycogen during resting conditions in the brain (Brown, 2004; Oz et al., 2007), but it can obviously be accelerated to underwrite memory processing in the hippocampus and cortex, as illustrated in **Figure 3**. DAB is only active in the brain for a short time period as when injected 10 min before training DAB had no effect on memory. In agreement with this idea, DAB injected 5 min prior to training memory is only shown to be effective when first tested 5 min after training, suggesting that it does not remain active in the brain for long and therefore only acts on memory processes very close to the time of injection, even though the consequences appear later.

Our data clearly show that glycogen has an active role in astrocytic function. This may in turn also influence neuronal metabolism because glycogenolysis may inhibit astrocytic glucose consumption and thereby make more glucose available for neurons (DiNuzzo et al., 2012, 2015). Since glycogen turnover is slow under resting conditions, the question arises as to how fast it can be recruited in cells and are there readily accessible stores with fast turnover in brain regions important for memory. It is possible that high turnover could occur in specific brain regions during neural activation. Glycogen phosphorylase can be activated within seconds and is stimulated during various behavioral activities (Walling et al., 2006). In chick astrocyte cultures, we have shown an initial decrease in glycogen levels after 5–10 min of incubation with noradrenaline, this decrease is then followed by an increase in glycogen levels (Hutchinson et al., 2011). The fall in glycogen levels, but not the later increase, also occurs with incubation with zinterol. These biochemical results suggest that the onset of breakdown of glycogen can occur quickly.

We designed experiments where the glycogen synthesis inhibitor ARC239 was challenged with zinterol (increasing glycogenolysis and promoting weakly reinforced learning) in two different paradigms (Gibbs and Hutchinson, 2012; **Figures 8B,C**) to ask two questions: firstly, can prevention of glycogen re-synthesis reduce glycogen levels sufficiently so that zinterol is unable to promote weakly reinforced learning, and at when does this occur after training? When we prevented glycogen synthesis by injecting ARC239, given 5 min before training, zinterol was able to promote consolidation given either immediately or 5 min after training, but it was unable to promote memory when given later than this, at 10, 15, or 20 min after training (**Figure 8B**), although zinterol will normally do so at these times. These results suggest that the ARC239-mediated inhibition following its injection 5 min before training took 10 min to reduce the readily available glycogen stores.

Secondly, we asked by what mechanisms and at what times can inhibition of glycogen synthesis affect the ability of zinterol to promote consolidation after weakly reinforced training? Zinterol was injected 20 min after training to promote consolidation and ARC239 at times between 5 min before and 15 min after training (i.e., 20–5 min before zinterol) prevented the β_2_-AR agonist promoting consolidation at all times except 5 min before training (**Figure 8C**). ARC239 given up to 10 min before zinterol prevented it from promoting consolidation. These results again suggest that depletion of accessible glycogen stores takes place quickly, i.e., within 10 min of injection.

## Serotonin and Glycogenolysis

Serotonin is another neuromodulatory transmitter that promotes memory consolidation (Gibbs and Hertz, 2014). Like noradrenaline, serotonin activates glycogenolysis in brain tissue and in astrocytes (Quach et al., 1982; Cambray-Deakin et al., 1988; Magistretti, 1988; Chen et al., 1995; Kong et al., 2002; Darvesh and Gudelsky, 2003). Although the β_2_-AR activation appears to be the signal leading to the second glycogenolytic period in IMM, noradrenaline is not implicated in the first period close to training. Serotonin has effects on memory in the chick dependent on the dose. Low doses of serotonin promote memory consolidation when injected up to 25 min after weakly reinforced training whereas high doses inhibit memory (Gibbs and Hertz, 2014). Serotonin thus appears to act on at least two different 5HT receptors during learning stimulating a high affinity serotonin receptor, identified below as the 5HT_2B_ receptor and inhibiting a lower-affinity serotonin receptor, possibly a 5-HT_1_ receptor.

**FIGURE 9:**
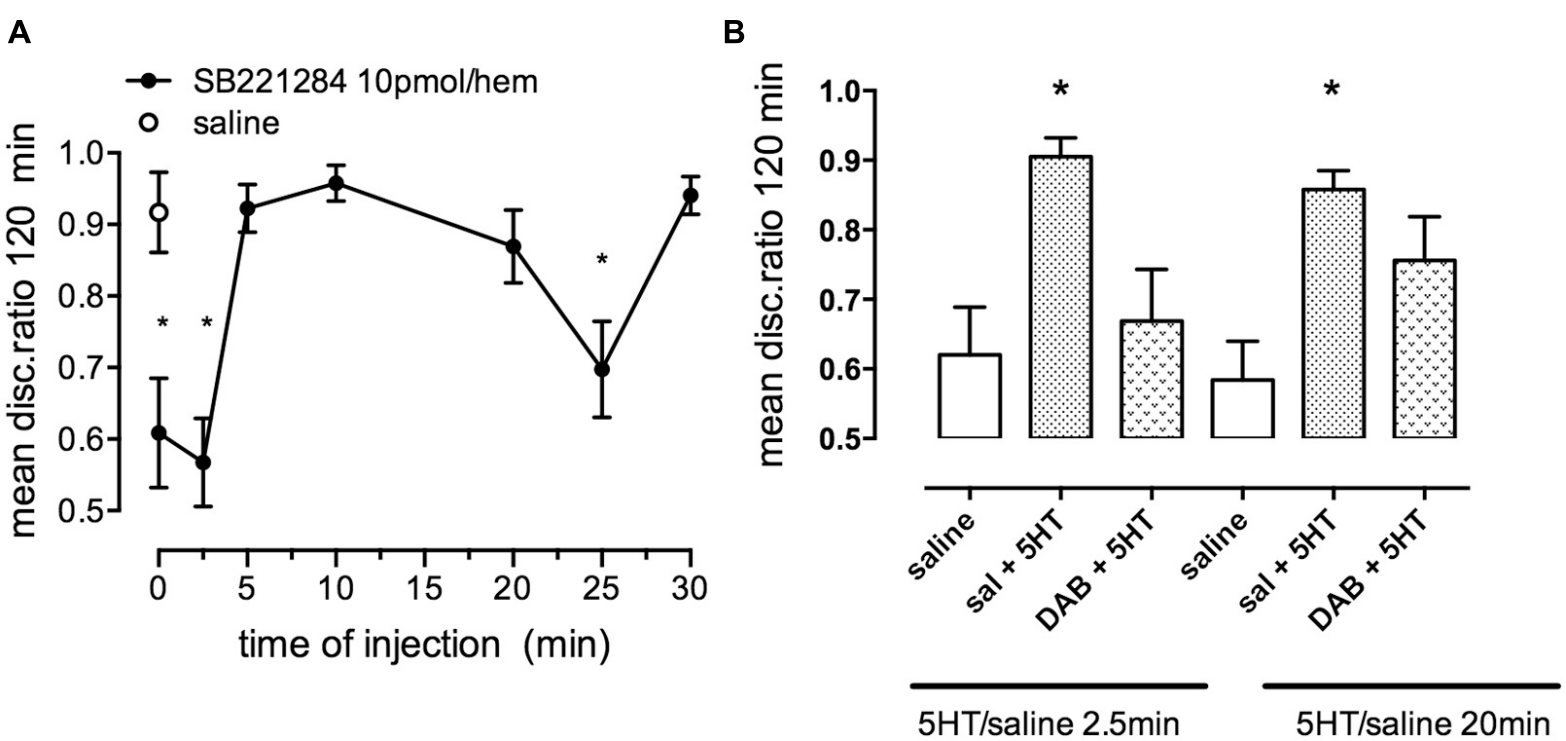
**Effect of the selective 5-HT_2B/C_ receptor antagonist SB221284 on strongly reinforced memory. (A)** Time of injection of SB221284 following strongly reinforced training. **(B)** Ability of the inhibitor DAB to prevent consolidation of weakly reinforced memory. A suboptimal dose of DAB was given either before or 15 min after training, serotonin was given 2.5 or 20 min after training. DAB only interfered with serotonin induced consolidation at the early period. ^*^P < 0.05. (From Gibbs and Hertz, 2014).

Strongly reinforced memory is inhibited by the selective 5HT_2B,C_ antagonist SB221284 injected immediately, 2.5 or 25 min after (**Figure 9A**). Conversely, weakly reinforced learning can be rescued by administration of serotonin (**Figure 9B**). In contrast to the β_2_-AR involvement, it is only during the first period where SB221284 inhibits (2.5 min after training) that the rescue by serotonin is challenged by a sub-optimal dose of DAB. In contrast, during the second glycogenolytic period where serotonin plays only a minor role, memory is not significantly affected when DAB is administered together with serotonin (**Figure 9B**). On the other hand the 5-HT_2B_ agonists fluoxetine and paroxetine (Li et al., 2008; Diaz et al., 2012) can consolidate weakly reinforced learning. It is interesting that fluoxetine and paroxetine which are better known as serotonin-specific antidepressants (SSRIs) also consolidate weakly reinforced learning in the chicken (Gibbs and Hertz, 2014). Fluoxetine also increases glycogenolysis in cultured astrocytes (Chen et al., 1995) by a 5-HT_2B_ receptor-mediated effect (Kong et al., 2002).

## ATP and Glycogenolysis

**FIGURE 10:**
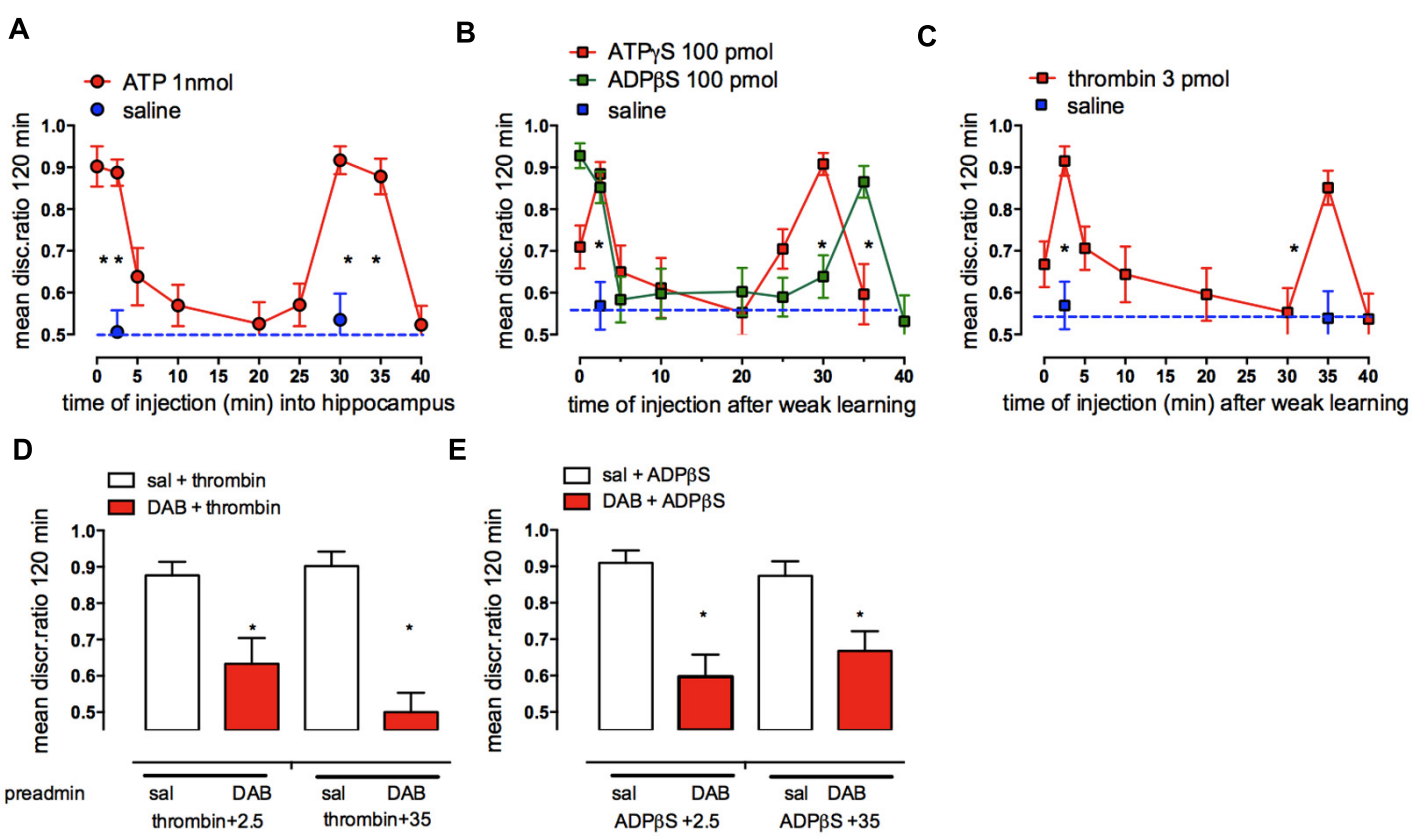
**Effect of hippocampal injection of ATP, ATPγS, ADPβS, or thrombin **(A–C)** on consolidation of weakly reinforced training and the effect of DAB on the ability of thrombin, ADPβS to promote consolidation **(D,E)**.** Injections over two time periods after weakly reinforced training resulted in consolidation of memory 120 min after training. **(A)** ATP, **(B)** ADPβS and ATPγS and **(C)** thrombin. **(D,E)** A sub-optimal dose of DAB (red columns) was injected subcutaneously 5 min before weakly reinforced training or 5 min before injection of thrombin or ADPβS into the hippocampus 2.5 or 35 min after weakly reinforced training, i.e., at times when they normally promote consolidation (open columns). ^*^P < 0.05. (From Gibbs et al., 2011).

As with the neurotransmitters, noradrenaline and serotonin, memory consolidation is also modulated by endogenous adenosine triphosphate (ATP) acting at purinergic receptors in the hippocampus (Gibbs et al., 2011) and in the cortex (IMM; Cronin et al., 2011). ATP is important for communication between neuronal and glial circuits and is released from both neurons and astrocytes (Fields and Burnstock, 2006; North and Verkhratsky, 2006). ATP released from astrocytes acts as a widespread gliotransmitter, triggering, and maintaining calcium signaling and calcium oscillations (Bowser and Khakh, 2004; Burnstock, 2007; Zorec et al., 2012). Release of transmitter ATP from cultured astrocytes is inhibited by DAB (Hertz et al., 2014b; Xu et al., 2014). On the other hand ATP is also known to trigger glycogenolysis (Hertz et al., 2015c). ATP injected into either the hippocampus (**Figure 10A**) or IMM promoted consolidation of weakly reinforced training at two time periods: 0–2.5 and 25–30 min post-training. The two non-hydrolyzable agonist analogs, ATPγS and ADPβS (**Figure 10B**) produced similar but not identical effects on memory time courses, in particular with ADPβS promoting consolidation at 35 but not at 30 min, whereas ATPγS promoted consolidation when injected at 30 but not at 35 min. Challenge to the action of ADPβS by the selective ADPβS antagonist MRS2179 showed that it acts via purinergic P_2_Y_1_ receptors (Gibbs et al., 2011).

Astrocytes are a major source of the released ATP in hippocampal slices in rodents (Bowser and Khakh, 2007), and evidence suggesting astrocytes are the source of the ATP promoting memory comes from the effect of thrombin on memory processing. Thrombin selectively activates calcium release from intracellular stores in astrocytes (Bowser and Khakh, 2004) and thrombin injected into IMM (Gibbs and Bowser, 2010) or the hippocampus (**Figure 10C**; Gibbs et al., 2011) like ADPβS, promoted memory consolidation when injected at 2.5 and 35 min after training. These results strongly suggest that astrocytes are the source of the ATP facilitating memory. Thrombin consolidation could also be successfully challenged by a low dose of the P2Y1 antagonist MRS2179. The inhibitory effect of fluoroacetate, which inhibits astrocytic oxidative metabolism, also confirms astrocytic involvement in promotion of memory by ATP (Gibbs and Bowser, 2009). Neither ADPβS nor thrombin were able to promote consolidation with prior administration of fluoroactate (Gibbs et al., 2011).

Further support for astrocytic involvement in purinergic memory formation comes from the successful challenge by DAB of both ADPβS and thrombin rescue of weakly reinforced learning (**Figures 10D,E**). DAB did not challenge ATPγS suggesting that the source of the ATP mimicked by ATPγS could therefore be neurons, which co-release ATP with glutamate or noradrenaline (Burnstock, 2007). Incubation of astrocytes with ATPγS or ADPβS resulted in an increase in intracellular calcium [Ca^2+^]_i,_ with the effect of ADPβS being blocked by fluoroacetate and also by the P2Y1 antagonist, again implicating a specific role for astrocytic P2Y1 receptors in the calcium response. We suggested that the source of the ATP itself acting on neural P2Y1 receptors is most likely astrocytes, since thrombin selectively increases [Ca^2+^]_i_ in astrocytes but not in neurons (Bowser and Khakh, 2004). Astrocytic [Ca^2+^]_i_ must accordingly play an important role in the consolidation of short-term to long-term memory through activation of astrocytic P2Y1 receptors (Gibbs et al., 2011). An increase in [Ca^2+^]_i_ is also a prerequisite for stimulation of glycogenolysis (Hertz et al., 2015c). An important pathway for entry of Ca^2+^ is store-operated Ca^2+^ channels, and store-operated Ca^2+^ entry (SOCE) triggers astrocytic glycogenolysis in cortical astrocyte cultures (Müller et al., 2014). Accordingly administration of DAB reduces the amount of Ca^2+^ loaded into the sarco/endoplasmic reticulum.

## Glycogen Facilitation of *De Novo* Synthesis of Glutamate Used for Memory Processing and Involvement with Astrocytic Na,K-Atpase Activity

It was shown earlier (**Figure 2**) that glycogenolysis is essential for the production of glutamate and thus also of its metabolite GABA, two important neurotransmitters. Glutamate is supplied by astrocytes to neurons, which are unable to synthesize glutamate *de novo* as will be discussed below. If the breakdown of glycogen in astrocytes is compromised, then so is neural transmission (Hertz et al., 2003; Hertz and Zielke, 2004; Sickmann et al., 2009). This process would require relatively rapid turnover of glycogen *in vivo.*

Astrocytic glycogenolysis supports increased glutamate and glutamine synthesis at both 5 min (**Figure 2**; Hertz et al., 2003; Gibbs et al., 2007) and probably also after 30 min after training. Both these times are consistent with the times at which glutamate is released in trained chickens (Daisley et al., 1998; Daisley and Rose, 2002). The reduction in glycogen levels in the chick forebrain coincides with a transient increase in glutamate in the left forebrain over the first 5 min after training, and since there is no concomitant decrease in aspartate or the glutamate precursor glutamine, it indicates *de novo* synthesis of glutamate (Hertz et al., 2003; Gibbs et al., 2007). The importance of glycogenolysis for glutamate synthesis has been confirmed in the rat (Sickmann et al., 2012).

**FIGURE 11:**
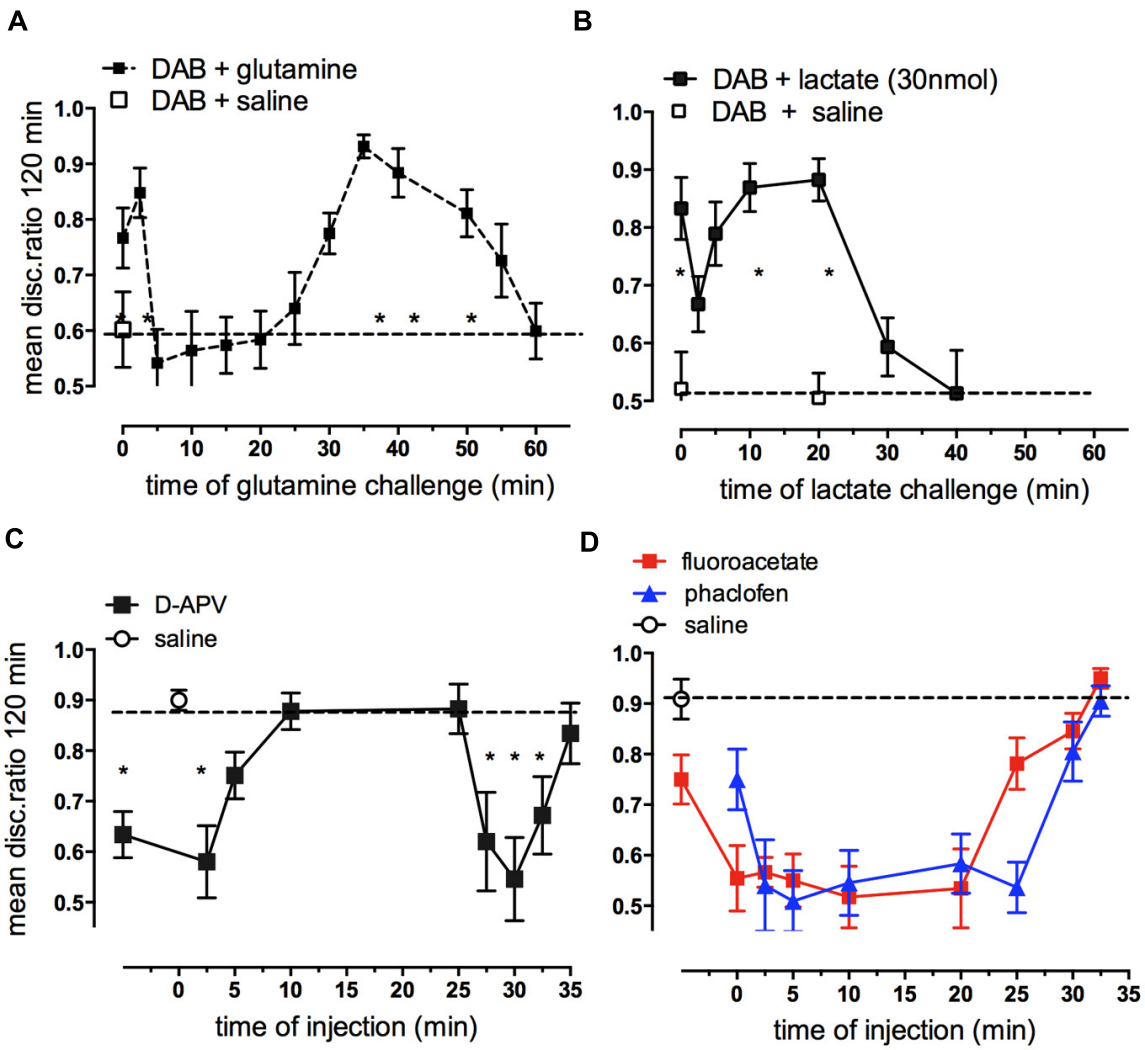
**Ability of glutamine or lactate to rescue memory following DAB inhibition of glycogenolysis **(A,B)** and effect of hippocampal injection of antagonists of NMDA receptors, GABA_B_ receptors or fluoroacetate on strongly reinforced training **(C,D)**.** DAB was injected into IMM 5 min before strongly reinforced training and challenged at various times by glutamine **(A)** or L-lactate **(B)** (From Hertz and Gibbs, 2009). **(C)** The NMDA angtagonist D-APV injected into the hippocampus inhibited memory given up to 5 min post-training and again when given 27.5–32.5 min post-training. **(D)** Both the GABA_B_ receptor antagonist, phaclofen and fluoroacetate inhibited memory when given between 2.5 and 25 min post-training. ^*^P < 0.05. (From Gibbs et al., 2008a; Gibbs and Bowser, 2009).

Since glycogen is responsible for the increased glutamate content in neurons occurring at specific times during memory consolidation, it would be expected that glutamine as a glutamate precursor should be able to rescue memory impairment caused by DAB. Our data show that glutamine can indeed rescue DAB-impaired memory when administered up to 2.5 min after training as well as during the second window for rescue 30–55 min after training. At times when DAB does not inhibit, i.e., when glycogen breakdown is not critical for memory consolidation (5–25 min) post-training glutamine does not rescue (**Figure 11A**) even though glutamine enhances weakly reinforced learning over this time period (5–30 min; Gibbs et al., 2006, 2007, 2008b). Additional experiments have shown that DAB is not successfully challenged by either aspartate or the astrocyte-specific metabolic substrate acetate alone. However, DAB-induced memory impairment can be rescued by acetate together with aspartate, with acetate needed as a precursor for the pyruvate carboxylase product oxaloacetate, because acetate alone cannot support pyruvate carboxylation (Gibbs et al., 2008b). These results suggest that a major function for glycogen during memory consolidation is to enable synthesis of glutamate and GABA via glutamate production in the astrocytes. This does not preclude that glycogen might also have a major role as a substrate for energy production. Moreover, both glutamate synthesis and its degradation are oxidative processes resulting in ATP production (Hertz et al., 2007). However, the relatively low rates of glycogenolysis in brain (Oz et al., 2012) should be kept in mind. Even the fast decline in glycogen occurring in the chick brain soon after training (Hertz et al., 2003) only amounts to ∼1.0 μmol/min per g wet wt, which is similar to the stimulated rate of glucose utilization in rat brain (Hertz and Dienel, 2002). A major role for glycogen might also be to support the energetic needs of astrocytes, including those related to their signaling (DiNuzzo et al., 2012; Xu et al., 2013). If the role of extracellular lactate is, indeed, a signaling one (as suggested above), then glycogenolysis might also promote signaling to neurons.

Lactate also rescues DAB induced impairment of memory when injected either immediately after training or 10–20 min later (**Figure 11B**). So, in contrast to the action of glutamine, lactate rescues memory from loss caused by DAB only at times when memory is NOT susceptible to DAB. These are the same time periods over which lactate normally enhances weakly reinforced learning (5–20 min; Gibbs et al., 2008b). Because of the difference in timing of the effect of lactate and glutamine, it is unlikely that they are producing the same end result of increasing glutamate. Lactate is readily taken up by neurons and astrocytes, but lactate uptake into astrocytes inhibits glycolysis (Hertz et al., 2014a). In that paper we discussed the fluxes of lactate into astrocytes, neurons, and between astrocytes and the multifunctional roles for lactate. Transport of lactate between brain cells is mainly between astrocytes via gap junctions and release into extracellular space leads to significant exit of lactate from the brain via the blood and via the perivascular-lymphatic drainage system (Ball et al., 2010).

Many years ago we found evidence that inhibition of the Na,K-ATPase abolished memory (Mark and Watts, 1971; Gibbs and Ng, 1977). Recent experiments have suggested that β_2_-adrenergic activity may partly alleviate the impaired memory in day-old chickens resulting from Na,K-ATPase inhibition (Hertz et al., 2015b). This would suggest involvement of the astrocytic Na,K-ATPase, which may require glycogenolysis for its function (DiNuzzo et al., 2010; Xu et al., 2013; Hertz et al., 2015b). Clearance of extracellular K^+^ increases signal to noise ratio and thus is likely to play an important role in synaptic plasticity (and hence in triggering memory formation).

## Glycogen as the Substrate for *De Novo* Synthesis of Glutamate and GABA

Both glutamate and GABA are involved in neurotransmission in the central nervous system and roles for glutamatergic NMDA (**Figure 11C**) and AMPA receptors (Gibbs et al., 2008a), mGluR and GABA_B_ (Gibbs and Bowser, 2009) receptors have been described for memory processing in the chick hippocampus. The importance of glutamate in memory formation up to 30 min post-training is highlighted by its effects at the two time periods 0–2.5 and 25–30min, i.e., during STM and at the ITM transition from ITMA to ITMB. ATP and thrombin are similarly involved at these two times (Gibbs et al., 2011). A recent report has shown that DAB inhibition of glycogenolysis prevents LTP beyond 30 min in the rat hippocampus (Suzuki et al., 2011). We have shown above that one important reason why consolidation of memory is dependent on glycogenolysis is because of the brain’s requirement for glutamate formation. However, both fluoroacetate (**Figure 11D**) and DAB (**Figure 3D**) injected into the hippocampus impair memory when given in between these times, implicating astrocytic activity and glycogenolysis over this period. The involvement of glycogen breakdown in memory is more extensive in the hippocampus than in the cortical IMM, and this may reflect on the requirement for astrocytes to supply GABA for interneurons. It is of interest that in IMM, fluoroacetate loses its inhibitory effect earlier than in the hippocampus and by 10 min after training it is ineffective in disrupting memory (Gibbs and Ng, 1977). GABA produced from glutamate is also dependent on glycogenolysis and inhibition of GABA_B_ receptors by the selective antagonist phaclofen resulted in memory inhibition injected at all times between 2.5 and 25 min after training (**Figure 11D**; Gibbs and Bowser, 2009).

## Why Astrocytes are Required for Glutamate Synthesis

**FIGURE 12:**
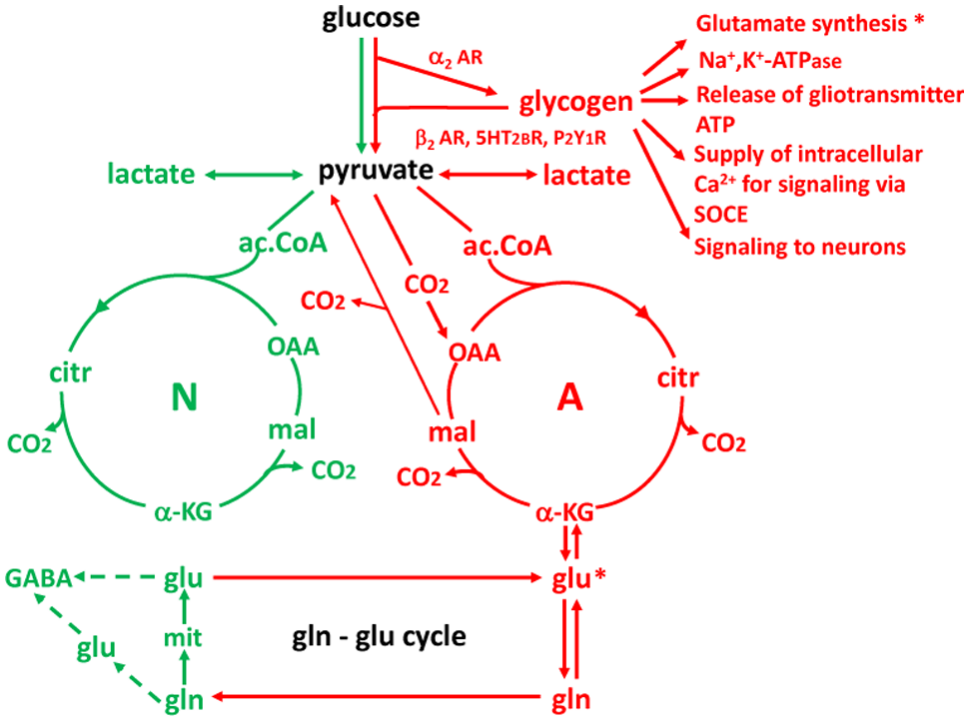
**Cartoon showing key features of glucose metabolism in neurons (N and green) and astrocytes (A and red) and interactions between the two cell types.** Three reactions of importance in the present review are astrocyte-specific: (1) glycogenolysis (and thus its stimulation by noradrenergic, serotonergic and purinergic subtype-specific agonists); (2) formation of the tricarboxylic acid (TCA) cycle intermediate oxaloacetate (OAA) by condensation of pyruvate with CO_2_ (pyruvate carboxylation); and (3) formation of glutamine from glutamate by glutamine synthetase. Reaction (1) is important for the transmitter actions on memory and for glutamate synthesis documented in this review, and for partial alleviation by β_2_-adrenergic activity of DAB-impaired memory resulting from Na,K-ATPase inhibition and inhibition of release of gliotransmitter ATP and of SOCE by DAB referred to. It is also important for release of lactate acting on neurons. Reaction (2) is essential for creation of a new molecule of a TCA cycle constituent OAA, allowing another molecule of a TCA cycle constituent, α-KG to leave the cycle and form glutamate. ^*^Indicates the importance of glycogenolysis for glutamate synthesis and glutamate synthesis from α-KG. And reaction (3) is a crucial step in supplying neurons with both newly synthesized and previously released glutamate. A fourth astrocyte-specific reaction is conversion of malate to pyruvate after its exit from the TCA cycle. This reaction allows metabolic degradation of accumulated glutamate but is not discussed in the review. The Figure should not give the impression that one half of brain energy metabolism is neuronal and one half astrocytic. Astrocytes account for a smaller fraction of the volume and therefore only for about one quarter of total glucose metabolism in gray matter (reviewed by Hertz, 2011), and other cell types like microglia also contribute to total energy metabolism.

The reason neurons are unable to synthetize glutamate (and thus also GABA) and therefore are dependent upon glycogenolysis-mediated glutamate synthesis is that they lack an enzyme, pyruvate carboxylase, which is present in astrocytes (Yu et al., 1983; Shank et al., 1985) and most cell types outside the brain. Pyruvate carboxylase activity is required for direct conversion of pyruvate to oxaloacetate (OAA), which in turn is needed for the astrocytic production of glutamate (**Figure 12**). Metabolism of pyruvate by the pathway via acetyl coenzyme A (ac.CoA) which is used for energy production does not allow production of a *new* tricarboxylic acid (TCA) constituent because the two carbon atoms which are introduced into the TCA cycle with ac.CoA are released (providing energy) during its subsequent turn to OAA. Such a new molecule of a TCA cycle constituent is necessary for glutamate formation because glutamate is formed from another TCA cycle constituent, α-ketoglutarate, α-KG, which accordingly is removed from the cycle.

After its formation in astrocytes glutamate is converted by the astrocyte-specific glutamine synthetase (Norenberg and Martinez-Hernandez, 1979; Anlauf and Derouiche, 2013) to glutamine which is released from astrocytes and accumulated into neurons by specific, regulated transporters (Nissen-Meyer and Chaudhry, 2013) in the glutamine-glutamate cycle (gln-glu cycle) and re-converted to glutamate and GABA (with slightly different pathways used in glutamatergic and GABA-ergic neurons). It should be emphasized that not all transmitter release depends on the *de novo* synthesis, since the transmitters are also re-utilized. However, especially glutamate is accumulated into astrocytes, not the neurons from which it was released, and again transferred to neurons after conversion to glutamine in astrocytes. This process accounts for ∼75% of the flux in the gln-glu cycle whereas the remaining ∼25% represents *de novo* synthesis (reviewed by Gibbs et al., 2008b; Hertz, 2013; Hertz and Rothman, 2015).

The similarly astrocyte-specific glycogenolysis (Pfeiffer-Guglielmi et al., 2003) is also shown in **Figure 12**, and its correlation with glutamine synthesis indicated by asterisks, although it is probably not glutamate formation from α-KG but rather pyruvate carboxylation that requires glycogenolysis. This review has documented the importance of glycogenolysis for both glutamate formation and Na,K-ATPase activity and references have been made to its importance for release of gliotransmitter ATP and for supply of intracellular Ca^2+^ by store-operated Ca^2+^ entry (SOCE). Similarly, the possibility that glycogen-derived lactate may be an important signal to neurons has been discussed. **Figure 12** summarizes these mechanisms (red text) as well as stimulation of glycogenolysis by β_2_-AR, 5-HT_2B_ receptors and P2Y1 receptors and of glycogen synthesis by α_2_AR, all documented in the review.

## Concluding Remarks

The excitatory and inhibitory neurotransmitters, glutamate, and GABA, are mainly responsible for information transfer and communication between nerve cells, but it is the modulation of synaptic activity by neurotransmitters including noradrenaline, serotonin and ATP that determines whether information in short-term or intermediate memory is consolidated into permanent storage or allowed to fade. These neuromodulatory transmitters act via astrocytes, stimulating the breakdown of glycogen that enables the synthesis of glutamate and glutamine that is essential for maintaining normal neuronal levels of glutamate and GABA and also for the regulation of potassium homeostasis (Hertz et al., 2015a).

Serotonin is responsible for the glycogen breakdown required for the transition from STM to ITM 2.5 min after training and noradrenaline for the transition from intermediate to long-term memory 30 min after training. Even if glycogen’s major role should be to provide energy, it clearly has another role in the brain where it is responsible for the synthesis of glutamine, the precursor for glutamate in neurons that are unable to synthesize glutamate *de novo* and most likely also for astrocytic K^+^ uptake (Xu et al., 2013; Hertz et al., 2015a), which constitutes an essential component of K^+^ homeostasis in the brain.

All noradrenergic input into the forebrain comes from cell bodies located in the locus coeruleus situated in the medulla. These fibres radiate out to brain areas including the hippocampus and the cortex where noradrenaline released acts on the noradrenergic receptors located on neurons, astrocytes, and microvessels and therefore can influence memory (Gibbs et al., 2010). The effect of noradrenaline on different cell types depends on the location of the different adrenergic receptors. The effects of noradrenergic stimulation on astrocytes, neurons and microglia, has been reviewed recently by O’Donnell et al. (2012).

In the avian cortex and hippocampus acts released noradrenaline acts on both β_2_ and α_2_ adrenergic receptors to cause the breakdown and the re-synthesis of glycogen. It promotes the transition of intermediate to long-term memory, whereas serotonin-induced glycogenolysis is responsible for the earlier consolidation of STM to intermediate memory, probably without any concomitant effect of serotonin on glycogen re-synthesis. This might explain why the level of glycogen is reduced 5 min after training (when serotonin mediates glycogenolysis) but not 30 min after training, when noradrenaline mediates glycogenolysis as well as glycogen synthesis (**Figure 3A**).

## Author Contributions

MG is the sole author of this review and which constitutes published work with a number of others.

## Conflict of Interest Statement

The author declares that the research was conducted in the absence of any commercial or financial relationships that could be construed as a potential conflict of interest.
